# A short-term, exploratory randomized controlled trial on the safety and feasibility of intraoperative raltitrexed peritoneal chemotherapy in laparoscopic radical resection for advanced colorectal cancer

**DOI:** 10.3389/fsurg.2026.1545705

**Published:** 2026-02-09

**Authors:** Haipeng Jin, Jun Yao, Zhiping Wei, Wenqiang Zhou, Chen Chen, Rongbiao Ying

**Affiliations:** 1 Department of Colorectal Surgery, Taizhou Cancer Hospital, Taizhou, China; 2 Graduate School, Zhejiang Chinese Medical University, Hangzhou, China; 3 Taizhou Key Laboratory of Minimally Invasive Interventional Therapy and Artificial Intelligence, Taizhou, China

**Keywords:** colorectal cancer, intraperitoneal perfusion chemotherapy, laparoscopic surgery, raltitrexed, safety

## Abstract

**Background:**

Patients with advanced colorectal cancer (CRC) with primary tumor stage T3 or T4 are at increased risk of peritoneal metastasis. The safety and feasibility of combining radical resection with intraoperative intraperitoneal perfusion chemotherapy (IPC) using raltitrexed in this population warrant further investigation.

**Methods:**

In this single-center, exploratory randomized controlled trial, 60 patients with advanced CRC (T3, T4) scheduled for laparoscopic radical resection were randomly assigned to receive either intraoperative raltitrexed IPC (*n* = 30) or surgery alone (control, *n* = 30). The primary endpoints were safety and feasibility. Toxicity profiles (hematologic, renal, hepatic), postoperative complications within 14 days, and procedural feasibility were compared between groups. Short-term tumor marker levels [carcinoembryonic antigen [CEA] and carbohydrate antigen 19-9 [CA19-9]] were assessed as exploratory endpoints, measured preoperatively and at three months postoperatively, with recurrence or metastasis events recorded within the same period.

**Results:**

The IPC procedure was successfully completed in all assigned patients, confirming procedural feasibility. No significant between-group differences were observed in postoperative hematological toxicity, nephrotoxicity, or complication rates. Transaminase elevations (ALT/AST) on postoperative day seven were transient and mild in both cohorts, with a more marked yet clinically manageable increase in the IPC group. At the three-month follow-up, no significant between-group differences were found in tumor marker levels or recurrence rates. Within-group analyses, however, demonstrated a significant decrease in CEA in the IPC group [mean change: −2.23 ± 4.68 ng/mL (95% CI: 0.48–3.98); *P* = 0.014] and a significant increase in CA19-9 in the control group [mean change: +3.33 ± 7.07 U/mL (95% CI: 0.69–5.97); *P* = 0.015].

**Conclusions:**

For patients with advanced CRC (T3/T4), laparoscopic radical resection combined with intraoperative raltitrexed IPC is feasible and exhibits an acceptable short-term safety profile. Observed short-term changes in tumor markers are considered exploratory findings and require validation in subsequent, large-scale prospective studies with extended follow-up periods.

## Introduction

Colorectal cancer (CRC) is a leading malignant tumor of the digestive system in China. According to the 2022 prevalence data released by the National Cancer Center, CRC accounts for approximately 517,000 new cases and 240,000 deaths annually, ranking second and fourth respectively among all malignancies (1). This epidemiological burden may be associated with shifts in population lifestyle and dietary patterns toward Westernized habits. Due to its insidious onset, aggressive behavior, and high risk of recurrence, a substantial proportion of patients are diagnosed at an advanced stage (T3 or T4). In T3–T4 CRC, tumor invasion extends into or beyond the intestinal serosa, increasing the risk of cancer cell detachment and subsequent peritoneal metastasis (PM). Epidemiological studies indicate that about 25% of CRC patients present with local implantation or distant metastasis at initial diagnosis. While the liver and lungs are the most common sites of distant metastasis, PM is detected in approximately 4%–8% of patients at diagnosis (2). Laparoscopic radical resection has become a standard surgical approach for CRC owing to its advantages such as enhanced visual field, minimal invasiveness, and quicker postoperative recovery. However, surgery alone cannot eliminate residual free cancer cells or microscopic tumor deposits within the abdominal cavity. Evidence suggests that PM significantly worsens prognosis after radical resection (3). Peritoneal dissemination occurs in nearly 17% of patients with metastatic CRC, and among those undergoing curative resection, 4%–19% develop metachronous PM during follow-up. Furthermore, isolated peritoneal dissemination is observed in about 2% of all CRC cases. Patients with peritoneal dissemination generally exhibit poorer outcomes compared to those without, with a median overall survival of only 6–9 months, inversely correlated with the extent of PM (4, 5).

The development of PM in CRC is fundamentally attributed to the presence of free cancer cells within the peritoneal cavity. According to the “seed-soil” theory, the interaction between disseminated tumor cells and the peritoneal surface creates a microenvironment that facilitates tumor cell colonization and proliferation (6). The progression of PM depends critically on this dynamic interplay between tumor cells and the peritoneal milieu. Tumor-derived cytokines contribute to the formation and remodeling of the extracellular matrix, thereby promoting the infiltration and adhesion of free cancer cells (7, 8). The pathways to PM are multifactorial. Firstly, from a pathological perspective, cancer cells from primary tumors—particularly in T3 and T4 stages—can penetrate the serosal layer and shed into the abdominal cavity, forming microscopic tumor deposits. The incidence of PM is significantly higher in T3–T4 cancers compared to T1–T2 lesions, with rates of abdominal implantation reaching up to 50% in T4 disease (9). Secondly, iatrogenic factors during surgery play a contributory role. Although laparoscopic radical resection for CRC offers advantages such as multi-angle visualization, minimal invasiveness, and faster recovery—making it a standard clinical procedure—the manipulation, squeezing, or traction of the tumor during resection may still cause tumor cell dislodgement. Furthermore, cutting tumor-associated blood or lymphatic vessels can lead to the spillage of blood or lymph into the peritoneal cavity, potentially carrying cancer cells. In addition, surgical techniques such as ultrasonic dissection can generate aerosols containing viable tumor cells; under conditions of CO₂ pneumoperitoneum and the “chimney effect”, these aerosols may increase the risk of implantation metastasis (10–12). Available data suggest that intraoperative peritoneal tumor dissemination may occur in up to 30% of CRC patients (13). Therefore, effectively eliminating or reducing free cancer cells and microscopic tumor deposits in the abdominal cavity after surgery is crucial for lowering recurrence rates and improving patient survival.

IPC is a regional treatment modality under clinical investigation for the management of PM, utilizing its distinct pharmacokinetic and hydrodynamic advantages. This modality aims to deliver high-dose, sustained local exposure of the peritoneal cavity to chemotherapeutic agents.

Currently, the use of IPC with raltitrexed in CRC remains underexplored. This exploratory study aimed to assess the safety and feasibility of combining raltitrexed IPC with laparoscopic radical resection in patients with advanced CRC, and to collect exploratory data on short-term tumor marker dynamics.

## Methods

### Study design and patients

This was a single-center, exploratory, randomized, single-blind, controlled trial conducted at Taizhou Cancer Hospital. The study was approved by the Hospital Ethics Committee (reference number: SL202320) and conducted in accordance with the Declaration of Helsinki (as revised in 2013). All patients or their guardians who participated in this study were sane and volunteered to be investigated, and written informed consent was obtained from all patients. Patients or their guardians had the right to withdraw from this study at any time for any reason.

The inclusion criteria were as follows: (I) colonoscopy and pathology confirmed the diagnosis of CRC; (II) CT or MR and other examinations suggested that the depth of tumor infiltration was T ≥ T3 or intraoperative confirmation that the tumor had invaded the plasma membrane layer of the intestinal wall or that the ascites was positive for exfoliated cells and that no metastasis to other organs had occurred; (III) aged 30–80 years with a Karnofsky score ≥70 and an expected survival period of ≥three months; (IV) preoperative white blood cell (WBC) count ≥3.0 × 10^9^/L, platelet (PLT) count ≥80 × 10^9^/L, and hemoglobin ≥80 g/L; and (V) signed an informed consent. The exclusion criteria were as follows: (I) those whose primary tumor foci could not be completely resected; (II) those with a preoperative combination of intestinal obstruction, intestinal perforation, abdominal infection, previous systemic chemotherapy or local radiotherapy; (III) those with severe chronic diseases or organ failure; (IV) those who could not tolerate the surgery and the treatment of abdominal chemotherapy; and (V) other patients judged by the responsible physician to be unsuitable for the trial.

As an exploratory safety and feasibility study, a formal sample size calculation based on efficacy outcomes was not conducted. A planned enrollment of 60 patients (30 per group) was considered adequate to generate preliminary safety profiles and evaluate procedural feasibility, in accordance with established guidelines for early-phase clinical investigations.

Eligible patients were randomized in a 1:1 ratio to either the IPC group or the control group. Randomization was performed using computer-generated block sequences (SPSS Statistics 27.0, IBM, USA) prepared by an independent researcher, with allocation concealment ensured through sealed, opaque envelopes.

### Investigation methods

All patients underwent laparoscopic radical colon cancer surgery or radical rectal cancer surgery performed by the same surgical team and were given 1,000 mL of warm distilled water to rinse the surgical wounds and the abdominal cavity repeatedly before abdominal closure, aspirating all the accumulated fluids, and leaving the abdominal drainage tube in place. In the treatment group (IPC group), after abdominal closure, a five millimeter trocar was used, the abdominal drainage tube was clamped and diluted, and 4 mg of raltitrexed (2 mg/strike, developed by Nanjing Chia-Tai Tianqing Pharmaceutical Company) was dissolved into 250 mL of 0.9% sodium chloride solution, which was rapidly dropped into the tumor resection site from the reserved poke card hole. The poke card was removed, the incision was closed, and the abdominal drainage tube was opened after 4 h. In the control group, routine abdominal closure and continuous opening of the abdominal drainage tube were performed.

The primary endpoints were safety and feasibility. Safety was assessed as follows: Peripheral blood samples were collected before and on postoperative day 7 to evaluate systemic toxicity. Hematologic toxicity [white blood cell [WBC] count, red blood cell [RBC] count, platelet [PLT] count], nephrotoxicity [serum creatinine [SCr], blood urea nitrogen [BUN]], hepatotoxicity [alanine aminotransferase [ALT], aspartate aminotransferase [AST]], and preoperative tumor marker levels (CEA, CA19-9) were recorded. Postoperative complications were documented through clinical observation for 14 days and included general symptoms [e.g., fever (defined as body temperature >38.5  °C for three consecutive days after surgery), nausea, vomiting, abdominal distension, diarrhea] and surgery-related complications (e.g., intra-abdominal hemorrhage, anastomotic leakage, intestinal obstruction, surgical site infection). Chemotherapy-related adverse events were graded according to the National Institutes of Health Common Terminology Criteria for Adverse Events version 3.0 (14). Feasibility was defined as the completion of the assigned treatment according to the protocol in both groups: specifically, the administration of IPC in addition to standard surgery in the intervention group, and the completion of standard surgery without protocol-defined deviations in the control group.

Patients were followed up every 3 months for the first 2 years postoperatively. At each visit, assessments included routine blood tests, liver and renal function panels, measurement of tumor markers (CEA, CA19-9), and digital rectal examination. Suspected recurrence, indicated by elevated tumor markers or findings on digital rectal examination, prompted further evaluation with contrast-enhanced CT of the entire abdomen. Patients with confirmed recurrence received further treatment—including reoperative intervention, systemic chemotherapy, or other appropriate antitumor therapies—in accordance with contemporary clinical guidelines. Exploratory endpoints included preoperative and 3-month postoperative levels of CEA and CA19-9, as well as the incidence of confirmed recurrence or metastasis within 3 months after surgery.

The clinical data of all the subjects were extracted from the database of the Taizhou Cancer Hospital medical records system.

### Statistical analysis

Statistical methods Data analysis was performed via SPSS statistics version 27.0. The categorical data are expressed in the form of numbers and percentages [*n* (%)] and were analyzed via Pearson's χ^2^ test and Fisher's exact test; the continuous data are expressed in the form of means ± standard deviations ($\bar{x}\pm \text{s}$) and were analyzed via Student's t test. Differences were considered statistically significant if *P* ≤ 0.05 in a two-tailed test with *α* = 0.05.

## Results

### Patient characteristics

A total of 60 patients with advanced CRC (T3, T4) who were admitted to the Department of Colorectal Surgery of Taizhou Cancer Hospital from February 2023 to August 2024 were included in this study. The average age was 64.80 ± 8.68 years. There were 37 males and 23 females, 22 with rectal cancer, 21 with sigmoid colon cancer, 3 with left colon cancer, 2 with transverse colon cancer and 12 with right colon cancer. There were 30 patients in the raltitrexed intraperitoneal perfusion group and 30 patients in the control group. All patients ultimately completed the main result analysis. There was no significant difference in age, sex, underlying disease, tumor location, T stage, N stage, pTNM stage or pathological type between the two groups (*P* > 0.05), and the baseline data were comparable (Table 1).

**Table 1:** Baseline characteristics of patients.

Variables	Treatment group (*N* = 30)	Control group (*N* = 30)	t/χ^2^ test	*P* value
Age (years)	64.70 ± 9.24	64.90 ± 8.24	0.09	0.93
Gender				
Male	19 (63.33)	18 (60.00)	0.07	0.79
Female	11 (36.67)	12 (40.00)		
High blood pressure				
No	24 (80.00)	19 (63.33)	2.05	0.15
Yes	6 (20.00)	11 (36.67)		
Type 2 diabetes				
No	26 (86.67)	24 (80.00)	0.48	0.49
Yes	4 (13.33)	6 (20.00)		
Tumor location				
Rectal	12 (40.00)	10 (33.33)	0.90	0.93
Mid rectal	8	4		
High rectal	4	6		
Sigmoid colon	11 (36.67)	10 (33.33)		
Left colon	1 (3.33)	2 (6.67)		
Transverse colon	1 (3.33)	1 (3.33)		
Right colon	5 (16.67)	7 (23.33)		
Tumor stage				
T3	23 (76.67)	21 (70.00)	0.34	0.56
T4	7 (23.33)	9 (30.00)		
Regional lymph node stage				
N0	15 (50.00)	13 (43.33)	1.01	0.60
N1	11 (36.67)	10 (33.33)		
N2	4 (13.33)	7 (23.33)		
pTNM stage				
ⅡA	13 (43.33)	11 (36.67)	2.81	0.73
ⅡB	1 (3.33)	2 (6.67)		
ⅡC	1 (3.33)	0 (0.00)		
ⅢA	1 (3.33)	9 (0.00)		
ⅢB	11 (36.67)	13 (43.33)		
ⅢC	3 (10.00)	4 (13.33)		
Degree of differentiation				
Highly differentiated	1 (3.33)	0 (0.00)	1.86	0.60
Moderately differentiated	14 (46.67)	11 (36.67)		
Medium-low differentiation	14 (46.67)	18 (60.00)		
Low differentiation	1 (3.33)	1 (3.33)		
Pathologic type				
Adenocarcinoma	27 (90.00)	26 (86.67)	0.00	>0.99
Mucinous adenocarcinoma	3 (10.00)	4 (13.33)		

### Systemic toxicity reactions and feasibility

The IPC procedure was successfully completed in all allocated patients, confirming feasibility. The differences in RBC, PLT, Cr and BUN between the two groups were not statistically significant (*P* > 0.05) at the preoperative and 7-day postoperative time points (Table 2). Compared with those in the preoperative period, the WBC, ALT and AST indices were greater in both groups at 7 days postsurgery, and the WBC [0.99 ± 2.48 (95% CI: 0.07–1.92) vs. 0.23 ± 1.58 (95% CI: −0.36–0.82)], ALT [25.44 ± 30.10 (95% CI:14.20–36.68) vs. 10.71 ± 20.83 (95% CI: 2.93–18.48)], AST [27.66 ± 31.10 (95% CI: 16.04–39.27) vs. 11.90 ± 25.12 (95% CI: 2.52–21.28)] were significantly greater in the treatment group than in the control group (*P* < 0.05) (Table 3).

**Table 2:** Comparison of the toxicity reactions of various systems before and after surgery in the two groups ($\bar{x}\pm \text{s}$).

Variables	Time	Treatment group (*N* = 30)	Control group (*N* = 30)	t test	*P* value
WBC (× 10^9^/L)	Preoperative	6.38 ± 1.40	5.92 ± 2.04	1.02	0.31
	7 days after operation	7.37 ± 2.56	6.15 ± 1.71	2.17	0.034
RBC (× 10^9^/L)	Preoperative	4.16 ± 0.57	4.07 ± 0.63	0.56	0.58
	7 days after operation	3.55 ± 0.59	3.74 ± 0.59	1.26	0.21
PLT (× 10^9^/L)	Preoperative	256.33 ± 84.53	238.73 ± 71.93	0.87	0.39
	7 days after operation	239.33 ± 86.85	216.40 ± 69.10	1.13	0.26
BUN (mmol/L)	Preoperative	4.52 ± 1.35	5.15 ± 1.63	1.63	0.11
	7 days after operation	5.39 ± 1.64	6.24 ± 1.71	1.95	0.06
SCr (μmol/L)	Preoperative	73.47 ± 16.10	82.97 ± 28.33	1.60	0.12
	7 days after operation	63.03 ± 12.83	71.03 ± 22.91	1.67	0.10
ALT (U/L)	Preoperative	13.72 ± 6.19	18.18 ± 9.65	2.13	0.038
	7 days after operation	39.16 ± 30.10	28.88 ± 20.08	1.56	0.13
AST (U/L)	Preoperative	19.62 ± 4.31	24.12 ± 9.98	2.27	0.029
	7 days after operation	47.27 ± 32.58	36.02 ± 24.31	1.52	0.14

**Table 3:** Comparison of the differences in WBC, ALT and AST levels before and after surgery between the two groups ($\bar{x}\pm \text{s}$).

Variables	Subgroup	Differentials (95% CI)	t test	*P* value
WBC (× 10^9^/L)	Treatment group	0.99 ± 2.48 (0.07–1.92)	2.19	0.037
	Control group	0.23 ± 1.58 (−0.36–0.82)	0.79	0.43
ALT (U/L)	Treatment group	25.44 ± 30.10 (14.20–36.68)	4.63	<0.001
	Control group	10.71 ± 20.83 (2.93–18.48)	2.82	0.009
AST (U/L)	Treatment group	27.66 ± 31.10 (16.04–39.27)	4.87	<0.001
	Control group	11.90 ± 25.12 (2.52–21.28)	2.59	0.015

Comparison with the preoperative cohort.

### Operative complications

The two groups of patients experienced different degrees of postoperative complications, including nausea and vomiting, abdominal distension and diarrhea, abdominal hemorrhage, anastomotic leakage, and intestinal obstruction staining, and the differences were not statistically significant (*P* > 0.05) (Table 4).

**Table 4:** Comparison of the occurrence of postoperative complications between the two groups of patients.

Variables	Treatment group (*n* = 30)	Control group (*n* = 30)	χ^2^ test	*P* value
Fever	0	0	0.00	>0.99
Nausea and vomiting	4	3	0.00	>0.99
Bloating	7	9	0.34	0.56
Diarrhea	1	0	0.00	>0.99
Abdominal bleeding	1	1	0.00	>0.99
Anastomotic leakage	2	1	0.00	>0.99
Bowel obstruction	2	0	0.52	0.47
Incision infection	0	0	0.00	>0.99

### Tumor markers and short-term recurrent metastasis

The differences in CEA, CA19-9 and recurrent metastasis between preoperative patients and 3-month postoperative patients and 3-month postoperative patients in the two groups were not statistically significant (*P* > 0.05). Compared with that before the operation, the CEA value of the treatment group significantly decreased by 2.23 ± 4.68 (95% CI: 0.48–3.98) at 3 months after the operation (t = 2.61, *P* = 0.014). In contrast, the CA19-9 value of the control group significantly increased by 3.33 ± 7.07 (95% CI: 0.69–5.97) at 3 months after the operation (t = 2.58, *P* = 0.015) (Table 5).

**Table 5:** Comparison of tumor marker levels before and after surgery and recurrent metastasis at 3 months after surgery between the two groups of patients.

Variables	Treatment group (*n* = 30)	Control group (*n* = 30)	t/χ^2^ text	*P* value
CEA (ng/mL)				
Preoperative	5.11 ± 4.77	3.41 ± 2.15	1.78	0.08
3 months after operation	2.88 ± 1.83	3.14 ± 1.55	0.60	0.55
Differentials (95% CI)	2.23 ± 4.68 (0.48–3.98)^a^	0.27 ± 2.04 (−0.50–1.03)^a^		
t text	2.61	0.72		
*P* value	0.014	0.48		
CA19-9 (U/mL)				
Preoperative	28.52 ± 79.22	14.58 ± 7.67	0.96	0.34
3 months after operation	21.25 ± 28.48	17.92 ± 11.57	0.59	0.56
Differentials (95% CI)	7.27 ± 57.92 (−14.35–28.90)^a^	−3.33 ± 7.07 (−5.97–0.69)^a^		
t text	0.69	2.58		
*P* value	0.50	0.015		
Recurrent metastasis	1	1	0.00	> 0.99

^a^ Compared with the same group 3 months after the operation.

## Discussion

This study analyzed the clinical data of 60 patients with locally advanced CRC (T3-T4). The results confirm that laparoscopic radical resection combined with intraoperative raltitrexed IPC is feasible and is associated with favorable short-term safety. This finding aligns with earlier reports by Adnan K. et al. (15, 16). Notably, the mean patient age in this study was approximately 65 years, which further supports the safety and tolerability of raltitrexed intraperitoneal perfusion in an elderly population.

IPC is a regional therapeutic modality under clinical investigation for PM, capitalizing on its distinct pharmacokinetic and hydrodynamic advantages. This modality is characterized by its capacity to achieve high-dose, sustained local exposure of the peritoneal cavity to chemotherapeutic agents. Preliminary evidence supports the further investigation of intraoperative IPC in patients with advanced CRC (9, 17). Furthermore, meta-analyses have evaluated the combination of cytoreductive surgery with intraoperative or early postoperative IPC regarding overall survival, in comparative analyses against control regimens (18, 19). 5-Fluorouracil (5-FU) is a conventional agent for IPC in CRC. It exerts its antitumor effect by inhibiting thymidylate synthase, thereby disrupting DNA synthesis. However, its short systemic half-life (approximately 20–30 min) may contribute to delayed recovery of bowel function and an increased risk of complications such as intestinal adhesions and abdominal infections. Raltitrexed is a quinazoline-based folate analog and a specific inhibitor of thymidylate synthase. Following active cellular uptake, raltitrexed is polyglutamated by folylpolyglutamate synthase. This polyglutamated form exhibits prolonged intracellular retention and more potent inhibition of thymidylate synthase, leading to sustained disruption of DNA synthesis. The long intracellular half-life (up to 198 h) of its polyglutamated derivatives sustains cytotoxic activity. Meta-analytic data suggest that raltitrexed may offer a favorable efficacy and toxicity profile compared to 5-FU in the context of colorectal cancer therapy (20, 21).

No significant differences were observed between the intervention and control groups in preoperative or postoperative day 7 levels of RBC, PLT, BUN, or SCr. In contrast, WBC, ALT, and AST levels increased from baseline in both groups at postoperative day 7. These increases were more pronounced in the intervention group, reaching statistical significance (*P* < 0.05). Notably, the mean increase in WBC count in the intervention group was 0.99 ± 2.48 × 10⁹/L (95% CI: 0.07–1.92), resulting in a mean absolute value of 7.37 ± 2.56 × 10⁹/L, which remained within the normal physiological range and did not necessitate clinical intervention. Collectively, these findings indicate that intraoperative raltitrexed IPC was not associated with clinically significant hematological or renal toxicity.

Postoperatively, elevations in ALT and AST levels were observed at day 7 in both groups, with a more pronounced increase in the intervention group compared to the control group. Specifically, the mean increases in ALT and AST from baseline in the intervention group were 25.44 ± 30.10 U/L (95% CI: 14.20–36.68) and 27.66 ± 31.10 U/L (95% CI: 16.04–39.27), respectively. In contrast, the corresponding increases in the control group were 10.71 ± 20.83 U/L (95% CI: 2.93–18.48) for ALT and 11.90 ± 25.12 U/L (95% CI: 2.52–21.28) for AST. This differential effect may be explained by the pharmacokinetics of intraperitoneal chemotherapy. Following intraperitoneal administration, a portion of the chemotherapeutic agent is absorbed via the peritoneum and enters the liver through the portal venous system, undergoing significant first-pass metabolism. This results in the liver being the primary site of high initial drug exposure (22). Simultaneously, the peritoneum-plasma barrier limits systemic absorption, thereby sustaining high local drug concentrations within the peritoneal cavity and, via portal circulation, in the liver and retroperitoneal region. These regional concentrations can reach 10–100 times those achieved with systemic intravenous administration, providing a pharmacological basis for localized effects, including potential hepatocellular stress (23). Furthermore, transient and reversible elevation of liver enzymes is a recognized class effect of many chemotherapeutic agents (24). In this study, the hepatic enzyme elevation associated with raltitrexed IPC was mild and self-limiting. Therefore, within the framework of regional chemotherapy, the higher enzyme levels in the IPC group may not merely represent toxicity, but could reflect the achievement of effective pharmacologic exposure within the target tissues (peritoneum and liver) (25). In this study, hepatoprotective therapy was initiated only for patients whose liver transaminases exceeded twice the upper limit of normal postoperatively. All such cases demonstrated normalization of liver enzymes within a short-term follow-up period.

Postoperative complications of varying severity were documented in both groups, including nausea, vomiting, abdominal distension, diarrhea, intra-abdominal bleeding, anastomotic leakage, and intestinal obstruction. Anastomotic leakage occurred in two patients in the intervention group, necessitating ileostomy, and in one patient in the control group, who was managed conservatively. These findings suggest that the intraoperative intraperitoneal administration of raltitrexed does not substantially increase the overall risk of postoperative complications.

In this study, tumor marker (CEA, CA19-9) levels were measured before surgery and at 3 months postoperatively, and recurrence/metastasis was tracked at the 3-month postoperative follow-up. No statistically significant intergroup differences were observed in 3-month postoperative CEA levels, CA19-9 levels, or the incidence of recurrence/metastasis. Further analysis revealed a significant decrease from baseline in CEA levels within the intervention group at 3 months (mean change: −2.23 ± 4.68 ng/mL; 95% CI: −3.98 to −0.48; *P* = 0.014). Conversely, a significant increase in CA19-9 levels was observed within the control group (mean change: +3.33 ± 7.07 U/mL; 95% CI: 0.69–5.97; *P* = 0.015). In this study, tumor markers were predefined as exploratory endpoints. Although within-group changes reached statistical significance, the absence of significant between-group differences, together with the limited follow-up period, precludes any assessment of clinical efficacy. These exploratory finding warrant further validation in larger prospective studies with extended follow-up.

There are several limitations of this study. First, this was a single-center study, and the sample size was small, which may introduce selection bias and affect the reliability of the statistical results. Second, the follow-up period was relatively short, and the effects on postoperative long-term survival, recurrence and metastasis have not yet been adequately investigated. Therefore, future studies may consider adopting increased sample sizes, prospective, randomized controlled, and multicenter cooperation in the clinical field, as well as extending the duration of patient follow-up. Long-term follow-up could provide valuable information on long-term patient outcomes and possible long-term side effects, adding new evidence to further validate the efficacy of raltitrexed IPC.

## Conclusions

In summary, intraoperative raltitrexed IPC combined with laparoscopic radical resection is feasible and safe in the short-term management of patients with advanced CRC (T3, T4), without augmenting surgical risks or major toxicity. Meanwhile, IPC is well tolerated by elderly patients, which further confirms that raltitrexed IPC has good safety and stability. Observed short-term changes in tumor markers are considered exploratory findings and necessitate validation in future, rigorously designed studies with sufficient statistical power and long-term oncological endpoints.

## Data Availability

The original contributions presented in the study are included in the article/Supplementary Material, further inquiries can be directed to the corresponding author.
